# Laryngology

**Published:** 1879-05

**Authors:** 


Dr. ELSBERG, of 614 Fifth Avenue, New York, is preparing for the American Laryngological Association a report of what has been published in this country on Laryngology and allied subjects; and will appreciate it as a favor if physicians will inform him of the title and publication of everything written on the subject of the Throat, Voice, etc. No matter how unimportant it may seem, please mention it in your answer.

